# The top 100 most cited articles in the past 30 years of wheat allergy: a bibliometric analysis

**DOI:** 10.3389/fimmu.2024.1381130

**Published:** 2024-04-22

**Authors:** Mengyuan Zhan, Yibo Hou, Liping Wen, Tengda Xu

**Affiliations:** ^1^ Department of Allergy, State Key Laboratory of Complex Severe and Rare Diseases, Peking Union Medical College Hospital, Chinese Academy of Medical Sciences and Peking Union Medical College, Beijing, China; ^2^ Allergy Department, Beijing Key Laboratory of Precision Medicine for Diagnosis and Treatment of Allergic Diseases, National Clinical Research Center for Dermatologic and Immunologic Diseases, Peking Union Medical College Hospital, Chinese Academy of Medical Sciences and Peking Union Medical College, Beijing, China; ^3^ Department of Health Care, Peking Union Medical College Hospital, Chinese Academy of Medical Sciences and Peking Union Medical College, Beijing, China

**Keywords:** wheat allergy, bibliometric analysis, diagnosis and treatment, wheat-dependent exercise-induced anaphylaxis, gliadin allergy, baker’s asthma

## Abstract

**Background:**

Wheat allergy (WA), characterized by immunological responses to wheat proteins, is a gluten-related disorder that has become increasingly recognized in recent years. Bibliometrics involves the quantitative assessment of publications within a specific academic domain.

**Objectives:**

We aimed to execute an extensive bibliometric study, focusing on the past 30 years of literature related to wheat allergy.

**Methods:**

We searched the Web of Science database on 5th Dec 2023. We used the keywords “wheat allergy or wheat anaphylaxis or wheat hypersensitivity,” “gliadin allergy or gliadin anaphylaxis or gliadin hypersensitivity,” “wheat-dependent exercise-induced anaphylaxis,” and “baker's asthma” for our search. All items published between 1993 and 2023 were included. The top 100 most cited articles were identified and analyzed.

**Results:**

Our study conducted an in-depth bibliometric analysis of the 100 most-cited articles in the field of wheat allergy, published between 2002 and 2019. These articles originated from 20 different countries, predominantly Japan and Germany. The majority of these articles were centered on the pathogenesis and treatment of wheat allergy (WA). The Journal of Allergy and Clinical Immunology (JACI) was the most prolific contributor to this list, publishing 14 articles. The article with the highest citation count was published by Biomed Central (BMC) and garnered 748 citations. The peak citation year was 2015, with a total of 774 citations, while the years 1998, 2001, and 2005 saw the highest publication frequency, each with 7 articles.

**Conclusion:**

Our study aims to provide physicians and researchers with a historical perspective for the scientific progress of wheat allergy, and help clinicians effectively obtain useful articles that have a significant impact on the field of wheat allergy.

## 1 Introduction

Wheat allergy manifests in diverse clinical forms. Among children, it often appears as typical symptoms of food allergy, marked by IgE-mediated responses, encompassing urticaria/angioedema and systemic allergic reactions. Over time, many patients experience a reduction in the severity of these symptoms, although complete tolerance is not consistently achieved across all cases (1). In adults, a significant manifestation is Wheat-Dependent Exercise-Induced Anaphylaxis (WDEIA), which presents as severe anaphylaxis triggered by the consumption of wheat, often in conjunction with physical activity or other exacerbating factors (2). Additionally, Baker’s asthma, a condition primarily affecting individuals in occupations with high exposure to flour dust, such as bakers, arises from the inhalation of wheat proteins (3).

Bibliometric analysis is a quantitative tool that employs statistical methods to assess the scope and impact of scientific literature, widely applied in fields such as Health Sciences and Medicine, Environmental Sciences, Engineering and Technology, and Social Sciences and Humanities. This method is instrumental in tracking research trends, influential authors, institutions, and publications, aiding in understanding the evolution of medical research and identifying key areas and knowledge gaps. In the context of food allergies, including wheat allergy, while the use of bibliometric analysis might not be as widespread as in other domains, it still plays a crucial role. It helps identify pivotal research articles, discern trends in allergy research, and uncover gaps in current understanding, which is especially valuable in dynamic fields like food allergies where new findings frequently emerge (4, 5).

In this study, we conducted a bibliometric analysis specifically focused on wheat allergy, aiming to fill a gap in the current research landscape. While previous bibliometric analyses have often concentrated on a broader spectrum of food allergies, studies specifically dissecting the domain of wheat allergy have been relatively scarce. Through an exhaustive search and rigorous selection of academic publications in the field of wheat allergy since 1993, we compiled comprehensive data encompassing publication trends, key research entities and authors, as well as prevalent research topics and directions that warrant further investigation. This analysis is intended to provide a holistic understanding of the historical development, current state, and future trajectory of wheat allergy research, laying the groundwork for subsequent discussions in this paper on the diagnosis, treatment, and management strategies of wheat allergy, as well as its impact on patient quality of life.

## 2 Methods

### 2.1 Data sources and search strategies

#### 2.1.1 Selection of database and rationale

In this bibliometric analysis, we opted for the Web of Science (WOS) Core Collection, guided by the comprehensive comparative study conducted by AlRyalat et al. (6).The pivotal factor influencing our decision was the unique ability of WOS to provide detailed and exhaustive descriptions of highly cited and hot papers, a feature not paralleled in alternative databases. This characteristic is crucial for accurate delineation and assessment of the most impactful literature in the field of wheat allergy research.

#### 2.1.2 Search strategy and criteria

The WOS Core Collection was meticulously searched on December 5, 2023. The search strategy encompassed a broad range of terms: “wheat allergy,” “wheat anaphylaxis,” “wheat hypersensitivity,” “gliadin allergy,” “gliadin anaphylaxis,” “gliadin hypersensitivity,” “wheat-dependent exercise-induced anaphylaxis,” and “baker’s asthma.” This comprehensive approach, spanning from 1993 to 2023, was designed to encapsulate the evolving landscape of research in this area. We imposed no constraints on document type or language, ensuring a wide-reaching and inclusive collection of data.

#### 2.1.3 Screening and inclusion process

The initial screening process was undertaken independently by two of our researchers, Mengyuan Zhan and Yibo Hou, who diligently reviewed abstracts to identify pertinent articles. Studies centralizing on wheat allergy (WA) or significantly involving WA were earmarked for inclusion. Conversely, articles tangential to WA were systematically excluded. This scrupulous process was conducted under the seasoned guidance of our senior experts, Liping Wen and Tengda Xu, culminating in a consensus on the top 100 most cited articles (T100). These articles represent the cornerstone of our bibliometric analysis, reflecting the most influential and pioneering work in the domain of wheat allergy research over the past three decades.

### 2.2 Data extraction and conversion

Data such as titles, authors, originating countries, affiliated institutions, publication years, publishing journals, types of articles, impact factors, and total number of citations was gathered from the WOS core database for analysis. The details of countries and institutions were determined based on the data from the primary corresponding author. The classification of article types included original research, review articles, clinical trials, clinical studies, and comments. The impact factors of journals were obtained from the 2023 Journal Citation Reports. It is widely recognized that citations are a key indicator of an article’s quality and impact. Nonetheless, it is important to note that it often takes time for an article to accumulate a significant number of citations post-publication. In our research, we employed the average citations per year (ACY) metric to mitigate this time-related bias, offering a more equitable comparison, especially for early-career academics. The ACY was calculated as follows: ACY = citation times/(2023-publication year+1).

### 2.3 Bibliometric analysis

In this study, the Bibliometrix R-package, a freely available open-source software, was employed for the quantitative analysis. Massimo Aria has highlighted that this R-package, a collaborative creation of Aria and Cuccurullo and written in the R programming language, is designed to facilitate bibliometric analysis for individuals without extensive coding knowledge (7). After installing R on our systems, we processed the data acquired from the Web of Science (WOS) in Plain Text format using the Biblioshiny application, a user-friendly interface equipped with an extensive range of statistical tools for analytical purposes.

Besides, this study classified the 100 cited articles into six distinct domains: ‘Clinical Manifestations,’ which delve into the signs and symptoms associated with wheat allergy; ‘Diagnostic Methods,’ concentrating on the array of techniques and procedures utilized in the diagnosis of wheat allergy; ‘Pathogenesis - Molecular Biology and Immunology of Wheat Allergens,’ encompassing research focused on the etiological pathways and molecular mechanisms underlying wheat allergies; ‘Epidemiology,’ analyzing the incidence, prevalence, and distribution patterns of wheat allergy within populations; ‘Prevention and Management,’ outlining the various strategies and therapeutic approaches in addressing wheat allergy; and ‘Others,’ a category reserved for studies that do not align neatly with the aforementioned domains.

## 3 Results

From the WOS Core Collection databases, a total of 2,800 documents were gathered, spanning the years 1993 to 2023. **Table 1** displays the top 100 most cited articles, organized in descending order by citation count. Regarding the study types in these top 100 (T100) articles on WA, 80% were article researches, 19% were reviews, and 1% were editorial materials.

**Table 1:** The top 100 cited articles on wheat allergy.

Rank	Title	Total Citations	Average per Year	Topic
1	Sapone A, Bai JC, Ciacci C, et al. Spectrum of gluten-related disorders: consensus on new nomenclature and classification [J]. BMC Med, 2012, 10: 12.	748	62.33	Epidemiology
2	Zuidmeer L, Goldhahn K, Rona RJ, et al. The prevalence of plant food allergies: A systematic review [J]. J Allergy Clin Immunol, 2008, 121: 1210-1218.	352	22	Epidemiology
3	Celik-Bilgili S, Mehl A, Verstege A, et al. The predictive value of specific immunoglobulin E levels in serum for the outcome of oral food challenges [J]. Clin Exp Allergy, 2005, 35: 268-273.	292	15.37	Diagnostic Methods
4	Tatham AS, Shewry PR. Allergens in wheat and related cereals [J]. Clin Exp Allergy, 2008, 38: 1712-1726.	251	15.69	Pathogenesis
5	Poole JA, Barriga K, Leung DYM, et al. Timing of initial exposure to cereal grains and the risk of wheat allergy [J]. Pediatrics, 2006, 117: 2175-2182.	230	12.78	Epidemiology
6	Weiss W, Vogelmeier C, Gorg A. electrophoretic characterization of wheat-grain allergens from different cultivars involved in bakers asthma [J]. Electrophoresis, 1993, 14: 805-816.	207	6.68	Pathogenesis
7	Palosuo K, Alenius H, Varjonen E, et al. A novel wheat gliadin as a cause of exercise-induced anaphylaxis [J]. J Allergy Clin Immunol, 1999, 103: 912-917.	189	7.56	Pathogenesis
8	Palosuo K, Varjonen E, Kekki OM, et al. Wheat ω-5 gliadin is a major allergen in children with immediate allergy to ingested wheat [J]. J Allergy Clin Immunol, 2001, 108: 634-638.	185	8.04	Diagnostic Methods
9	Hischenhuber C, Crevel R, Jarry B, et al. Review article:: safe amounts of gluten for patients with wheat allergy or coeliac disease [J]. Aliment Pharmacol Ther, 2006, 23: 559-575.	177	9.83	Epidemiology and Prevention and Management
10	Matsuo H, Morimoto K, Akaki T, et al. Exercise and aspirin increase levels of circulating gliadin peptides in patients with wheat-dependent exercise-induced anaphylaxis [J]. Clin Exp Allergy, 2005, 35: 461-466.	172	9.05	Pathogenesis
11	Eigenmann PA, Sampson HA. Interpreting skin prick tests in the evaluation of food allergy in children [J]. Pediatr Allergy Immunol, 1998, 9: 186-191.	171	6.58	Diagnostic Methods
12	Morita E, Kunie K, Matsuo H. Food-dependent exercise-induced anaphylaxis [J]. J Dermatol Sci, 2007, 47: 109-117.	170	10	Diagnostic Methods and Pathogenesis
13	Pastorello EA, Farioli L, Conti A, et al. Wheat IgE-mediated food allergy in European patients:: α-amylase inhibitors, lipid transfer proteins and low-molecular-weight glutenins -: Allergenic molecules recognized by double-blind, placebo-controlled food challenge [J]. Int Arch Allergy Immunol, 2007, 144: 10-22.	167	9.82	Pathogenesis
14	Armentia A, Díaz-Perales A, Castrodeza J, et al. Why can patients with baker’s asthma tolerate wheat flour ingestion? Is wheat pollen allergy relevant? [J]. Allergol Immunopath, 2009, 37: 203-204.	158	10.53	Pathogenesis
15	Dupont FM, Vensel WH, Tanaka CK, et al. Deciphering the complexities of the wheat flour proteome using quantitative two-dimensional electrophoresis, three proteases and tandem mass spectrometry [J]. Proteome Sci, 2011, 9: 29.	157	12.08	Epidemiology and Diagnostic Methods
16	Jones SM, Magnolfi CF, Cooke SK, et al. IMMUNOLOGICAL CROSS-REACTIVITY AMONG CEREAL-GRAINS AND GRASSES IN CHILDREN WITH FOOD HYPERSENSITIVITY [J]. J Allergy Clin Immunol, 1995, 96: 341-351.	155	5.34	Epidemiology and Diagnostic Methods
17	Wölbing F, Fischer J, Köberle M, et al. About the role and underlying mechanisms of cofactors in anaphylaxis [J]. Allergy, 2013, 68: 1085-1092.	152	13.82	Pathogenesis and Epidemiology
18	Keet CA, Matsui EC, Dhillon G, et al. The natural history of wheat allergy [J]. Ann Allergy Asthma Immunol, 2009, 102: 410-415.	151	10.07	Epidemiology
19	Matsuo H, Morita E, Tatham AS, et al. Identification of the IgE-binding epitope in ω-5 gliadin, a major allergen in wheat-dependent exercise-induced anaphylaxis [J]. J Biol Chem, 2004, 279: 12135-12140.	141	7.05	Pathogenesis
20	Elli L, Branchi F, Tomba C, et al. Diagnosis of gluten related disorders: Celiac disease, wheat allergy and non-celiac gluten sensitivity [J]. World J Gastroenterol, 2015, 21: 7110-7119.	138	15.33	Epidemiology and Diagnostic Methods
21	Battais F, Mothes T, Moneret-Vautrin DA, et al. Identification of IgE-binding epitopes on gliadins for patients with food allergy to wheat [J]. Allergy, 2005, 60: 815-821.	137	7.21	Pathogenesis
22	Harada S, Horikawa T, Ashida M, et al. Aspirin enhances the induction of type I allergic symptoms when combined with food and exercise in patients with food-dependent exercise-induced anaphylaxis [J]. Br J Dermatol, 2001, 145: 336-339.	137	5.96	Pathogenesis
23	Matsuo H, Kohno K, Niihara H, et al. Specific IgE determination to epitope peptides of ω-5 gliadin and high molecular weight glutenin subunit is a useful tool for diagnosis of wheat-dependent exercise-induced anaphylaxis [J]. J Immunol, 2005, 175: 8116-8122.	135	7.11	Diagnostic Methods and Pathogenesis
24	Brockow K, Kneissl D, Valentini L, et al. Using a gluten oral food challenge protocol to improve diagnosis of wheat-dependent exercise-induced anaphylaxis [J]. J Allergy Clin Immunol, 2015, 135: 977-+.	134	14.89	Epidemiology and Pathogenesis
25	Scherf KA, Koehler P, Wieser H. Gluten and wheat sensitivities - An overview [J]. J Cereal Sci, 2016, 67: 2-11.	133	16.63	others
26	Balakireva AV, Zamyatnin AA. Properties of Gluten Intolerance: Gluten Structure, Evolution, Pathogenicity and Detoxification Capabilities [J]. Nutrients, 2016, 8: 27.	132	16.5	Pathogenesis
27	Houba R, Heederik D, Doekes G. Wheat sensitization and work-related symptoms in the baking industry are preventable - An epidemiologic study [J]. Am J Respir Crit Care Med, 1998, 158: 1499-1503.	130	5	Epidemiology
28	Aihara M, Miyazawa M, Osuna H, et al. Food-dependent exercise-induced anaphylaxis: influence of concurrent aspirin administration on skin testing and provocation [J]. Br J Dermatol, 2002, 146: 466-472.	127	5.77	Diagnostic methods and Pathogenesis
29	Palosuo K, Varjonen E, Nurkkala J, et al. Transglutaminase-mediated cross-linking of a peptic fraction of ω-5 gliadin enhances IgE reactivity in wheat-dependent, exercise-induced anaphylaxis [J]. J Allergy Clin Immunol, 2003, 111: 1386-1392.	126	6	Pathogenesis
30	Schalk K, Lexhaller B, Koehler P, et al. Isolation and characterization of gluten protein types from wheat, rye, barley and oats for use as reference materials [J]. PLoS One, 2017, 12: 20.	124	17.71	Pathogenesis
31	Palosuo K. Update on wheat hypersensitivity [J]. Curr Opin Allergy Clin Immunol, 2003, 3: 205-209.	122	5.81	Clinical Manifestations、Pathogenesis、Epidemiology
32	Inomata N. Wheat allergy [J]. Curr Opin Allergy Clin Immunol, 2009, 9: 238-243.	121	8.07	Clinical Manifestations、Pathogenesis、Epidemiology
33	Sander I, Flagge A, Merget R, et al. Identification of wheat floor allergens by means of 2-dimensional immunoblotting [J]. J Allergy Clin Immunol, 2001, 107: 907-913.	119	5.17	Pathogenesis
34	Buchanan BB, Adamidi C, Lozano RM, et al. Thioredoxin-linked mitigation of allergic responses to wheat [J]. Proc Natl Acad Sci U S A, 1997, 94: 5372-5377.	119	4.41	Pathogenesis
35	Palacin A, Quirce S, Armentia A, et al. Wheat lipid transfer protein is a major allergen associated with baker’s asthma [J]. J Allergy Clin Immunol, 2007, 120: 1132-1138.	117	6.88	Pathogenesis
36	Matsuo H, Dahlström J, Tanaka A, et al. Sensitivity and specificity of recombinant ω-5 gliadin-specific IgE measurement for the diagnosis of wheat-dependent exercise-induced anaphylaxis [J]. Allergy, 2008, 63: 233-236.	115	7.19	Diagnostic methods
37	Battais F, Pineau F, Popineau Y, et al. Food allergy to wheat: identification of immunogloglin E and immunoglobulin G-binding proteins with sequential extracts and purified proteins from wheat flour [J]. Clin Exp Allergy, 2003, 33: 962-970.	114	5.43	Pathogenesis
38	Salcedo G, Quirce S, Diaz-Perales A. Wheat Allergens Associated With Baker’s Asthma [J]. J Invest Allergol Clin Immunol, 2011, 21: 81-92.	111	8.54	Diagnostic methods
39	James JM, Sixbey JP, Helm RM, et al. Wheat alpha-amylase inhibitor: A second route of allergic sensitization [J]. J Allergy Clin Immunol, 1997, 99: 239-244.	111	4.11	Diagnostic methods
40	Scherf KA, Brockow K, Biedermann T, et al. Wheat-dependent exercise-induced anaphylaxis [J]. Clin Exp Allergy, 2016, 46: 10-20.	110	13.75	Pathogenesis
41	Hill ID, Fasano A, Guandalini S, et al. NASPGHAN Clinical Report on the Diagnosis and Treatment of Gluten-related Disorders [J]. J Pediatr Gastroenterol Nutr, 2016, 63: 156-165.	109	13.63	Clinical Manifestationst
42	Baur X, Degens PO, Sander I. Baker’s asthma: Still among the most frequent occupational respiratory disorders [J]. J Allergy Clin Immunol, 1998, 102: 984-997.	108	4.15	others
43	Remington BC, Westerhout J, Meima MY, et al. Updated population minimal eliciting dose distributions for use in risk assessment of 14 priority food allergens [J]. Food Chem Toxicol, 2020, 139: 8.	107	26.75	Clinical Manifestations
44	Naqash F, Gani A, Gani A, et al. Gluten-free baking: Combating the challenges - A review [J]. Trends Food Sci Technol, 2017, 66: 98-107.	107	15.29	Diagnostic methods
45	Cullinan P, Cook A, Nieuwenhuijsen MJ, et al. Allergen and dust exposure as determinants of work-related symptoms and sensitization in a cohort of flour-exposed workers; a case-control analysis [J]. Ann Occup Hyg, 2001, 45: 97-103.	106	4.61	Epidemiology
46	Tanabe S, Arai S, Yanagihara Y, et al. A major wheat allergen has a Gln-Gln-Gln-Pro-Pro motif identified as an IgE-binding epitope [J]. Biochem Biophys Res Commun, 1996, 219: 290-293.	104	3.71	Pathogenesis
47	Akagawa M, Handoyo T, Ishii T, et al. Proteomic analysis of wheat flour allergens [J]. J Agric Food Chem, 2007, 55: 6863-6870.	101	5.94	Pathogenesis
48	Shewry PR. Do ancient types of wheat have health benefits compared with modern bread wheat? [J]. J Cereal Sci, 2018, 79: 469-476.	99	16.5	Epidemiology
49	Sandiford CP, Tatham AS, Fido R, et al. Identification of the major water/salt insoluble wheat proteins involved in cereal hypersensitivity [J]. Clin Exp Allergy, 1997, 27: 1120-1129.	99	3.67	Pathogenesis
50	Laurière M, Pecquet C, Bouchez-Magiout I, et al. Hydrolysed wheat proteins present in cosmetics can induce immediate hypersensitivities [J]. Contact Dermatitis, 2006, 54: 283-289.	98	5.44	Pathogenesis
51	Juhasz A, Belova T, Florides CG, et al. Genome mapping of seed-borne allergens and immunoresponsive proteins in wheat [J]. Sci Adv, 2018, 4: 15.	97	16.17	Epidemiology
52	Golley S, Corsini N, Topping D, et al. Motivations for avoiding wheat consumption in Australia: results from a population survey [J]. Public Health Nutr, 2015, 18: 490-499.	97	10.78	Pathogenesis
53	Constantin C, Quirce S, Poorafshar M, et al. Micro-arrayed wheat seed and grass pollen allergens for component-resolved diagnosis [J]. Allergy, 2009, 64: 1030-1037.	93	6.2	Pathogenesis
54	Weichel M, Glaser AG, Ballmer-Weber BK, et al. Wheat and maize thioredoxins: A novel cross-reactive cereal allergen family related to baker’s asthma [J]. J Allergy Clin Immunol, 2006, 117: 676-681.	93	5.17	Diagnostic methods
55	Morita E, Matsuo H, Mihara S, et al. Fast ω-gliadin is a major allergen in wheat-dependent exercise-induced anaphylaxis [J]. J Dermatol Sci, 2003, 33: 99-104.	93	4.43	Pathogenesis
56	Bunyavanich S, Rifas-Shiman SL, Platts-Mills TA, et al. Peanut, milk, and wheat intake during pregnancy is associated with reduced allergy and asthma in children [J]. J Allergy Clin Immunol, 2014, 133: 1373-1382.	92	9.2	Clinical Manifestations
57	Sander I, Rozynek P, Rihs HP, et al. Multiple wheat flour allergens and cross-reactive carbohydrate determinants bind IgE in baker’s asthma [J]. Allergy, 2011, 66: 1208-1215.	92	7.08	Epidemiology
58	Majamaa H, Moisio P, Holm K, et al. Wheat allergy: diagnostic accuracy of skin prick and patch tests and specific IgE [J]. Allergy, 1999, 54: 851-856.	88	3.52	Diagnostic methods
59	Mehl A, Verstege A, Staden U, et al. Utility of the ratio of food-specific IgE/total IgE in predicting symptomatic food allergy in children [J]. Allergy, 2005, 60: 1034-1039.	85	4.47	Diagnostic methods
60	Palosuo K, Alenius H, Varjonen E, et al. Rye γ-70 and γ-35 secalins and barley γ-3 hordein cross-react with ω-5 gliadin, a major allergen in wheat-dependent, exercise-induced anaphylaxis [J]. Clin Exp Allergy, 2001, 31: 466-473.	85	3.7	Pathogenesis
61	Matsuo H, Kohno K, Morita E. Molecular cloning, recombinant expression and IgE-binding epitope of ω-5 gliadin, a major allergen in wheat-dependent exercise-induced anaphylaxis [J]. Febs J, 2005, 272: 4431-4438.	82	4.32	Diagnostic methods
62	SanchezMonge R, GarciaCasado G, LopezOtin C, et al. Wheat flour peroxidase is a prominent allergen associated with baker’s asthma [J]. Clin Exp Allergy, 1997, 27: 1130-1137.	82	3.04	Pathogenesis
63	Simonato B, Pasini G, Giannattasio M, et al. Food allergy to wheat products: The effect of bread baking and *in vitro* digestion on wheat allergenic proteins. A study with bread dough, crumb, and crust [J]. J Agric Food Chem, 2001, 49: 5668-5673.	81	3.52	Pathogenesis
64	Hanakawa Y, Tohyama M, Shirakata Y, et al. Food-dependent exercise-induced anaphylaxis: a case related to the amount of food allergen ingested [J]. Br J Dermatol, 1998, 138: 898-900.	79	3.04	Clinical Manifestations
65	Brant A. Baker’s asthma [J]. Curr Opin Allergy Clin Immunol, 2007, 7: 152-155.	78	4.59	Epidemiology
66	Dezotti R, Larese F, Bovenzi M, et al. ALLERGIC AIRWAY DISEASE IN ITALIAN BAKERS AND PASTRY MAKERS [J]. Occup Environ Med, 1994, 51: 548-552.	77	2.57	Pathogenesis
67	Baur X, Posch A. Characterized allergens causing bakers’ asthma [J]. Allergy, 1998, 53: 562-566.	76	2.92	Epidemiology
68	Maruyama N, Ichise K, Katsube T, et al. Identification of major wheat allergens by means of the <i>Escherichia coli</i> expression system [J]. Eur J Biochem, 1998, 255: 739-745.	75	2.88	Pathogenesis
69	Hofmann SC, Fischer J, Eriksson C, et al. IgE detection to α/β/γ-gliadin and its clinical relevance in wheat-dependent exercise-induced anaphylaxis [J]. Allergy, 2012, 67: 1457-1460.	73	6.08	Diagnostic methods
70	Battais F, Courcoux P, Popineau Y, et al. Food allergy to wheat: differences in immunoglobulin E-binding proteins as a function of age or symptoms [J]. J Cereal Sci, 2005, 42: 109-117.	72	3.79	Pathogenesis
71	Kucek LK, Veenstra LD, Amnuaycheewa P, et al. A Grounded Guide to Gluten: How Modern Genotypes and Processing Impact Wheat Sensitivity [J]. Compr Rev Food Sci Food Saf, 2015, 14: 285-302.	71	7.89	Pathogenesis
72	Sealey-Voyksner JA, Khosla C, Voyksner RD, et al. Novel aspects of quantitation of immunogenic wheat gluten peptides by liquid chromatography-mass spectrometry/mass spectrometry [J]. J Chromatogr A, 2010, 1217: 4167-4183.	71	5.07	Diagnostic methods
73	Mittag D, Niggemann B, Sander I, et al. Immunoglobulin E-reactivity of wheat-allergic subjects (baker’s asthma, food allergy, wheat-dependent, exercise-induced anaphylaxis) to wheat protein fractions with different solubility and digestibility [J]. Mol Nutr Food Res, 2004, 48: 380-389.	71	3.55	Diagnostic methods
74	Christensen MJ, Eller E, Mortz CG, et al. Exercise Lowers Threshold and Increases Severity, but Wheat-Dependent, Exercise-Induced Anaphylaxis Can Be Elicited at Rest [J]. J Allergy Clin Immunol-Pract, 2018, 6: 514-520.	70	11.67	Diagnostic methods
75	Chinuki Y, Morita E. Wheat-Dependent Exercise-Induced Anaphylaxis Sensitized with Hydrolyzed Wheat Protein in Soap [J]. Allergol Int, 2012, 61: 529-537.	69	5.75	Clinical Manifestations
76	Scibilia J, Pastorello EA, Zisa G, et al. Wheat allergy: A double-blind, placebo-controlled study in adults [J]. J Allergy Clin Immunol, 2006, 117: 433-439.	69	3.83	Diagnostic methods
77	Amano M, Ogawa H, Kojima K, et al. Identification of the major allergens in wheat floor responsible for baker’s asthma [J]. Biochem J, 1998, 330: 1229-1234.	68	2.62	Pathogenesis
78	Christensen MJ, Eller E, Mortz CG, et al. Wheat-Dependent Cofactor-Augmented Anaphylaxis: A Prospective Study of Exercise, Aspirin, and Alcohol Efficacy as Cofactors [J]. J Allergy Clin Immunol-Pract, 2019, 7: 114-121.	67	13.4	Clinical Manifestations
79	Sotkovsky P, Hubálek M, Hernychová L, et al. Proteomic analysis of wheat proteins recognized by IgE antibodies of allergic patients [J]. Proteomics, 2008, 8: 1677-1691.	67	4.19	Pathogenesis
80	De Zotti R, Bovenzi M. Prospective study of work related respiratory symptoms in trainee bakers [J]. Occup Environ Med, 2000, 57: 58-61.	67	2.79	Epidemiology
81	Varjonen E, Vainio E, Kalimo K. Life-threatening, recurrent anaphylaxis caused by allergy to gliadin and exercise [J]. Clin Exp Allergy, 1997, 27: 162-166.	67	2.48	Pathogenesis
82	Weiss W, Huber G, Engel KH, et al. Identification and characterization of wheat grain albumin/globulin allergens [J]. Electrophoresis, 1997, 18: 826-833.	66	2.44	Diagnostic methods
83	Ito K, Futamura M, Borres MP, et al. IgE antibodies to ω-5 gliadin associate with immediate symptoms on oral wheat challenge in Japanese children [J]. Allergy, 2008, 63: 1536-1542.	65	4.06	Diagnostic methods
84	Simonato B, De Lazzari F, Pasini G, et al. IgE binding to soluble and insoluble wheat flour proteins in atopic and non-atopic patients suffering from gastrointestinal symptoms after wheat ingestion [J]. Clin Exp Allergy, 2001, 31: 1771-1778.	65	2.83	Diagnostic methods
85	Watanabe M, Watanabe J, Sonoyama K, et al. Novel method for producing hypoallergenic wheat flour by enzymatic fragmentation of the constituent allergens and its application to food processing [J]. Biosci Biotechnol Biochem, 2000, 64: 2663-2667.	62	2.58	prevention and management
86	Gil-Humanes J, Pistón F, Altamirano-Fortoul R, et al. Reduced-Gliadin Wheat Bread: An Alternative to the Gluten-Free Diet for Consumers Suffering Gluten-Related Pathologies [J]. PLoS One, 2014, 9: 9.	61	6.1	prevention and management
87	Bittner C, Grassau B, Frenzel K, et al. Identification of wheat gliadins as an allergen family related to baker’s asthma [J]. J Allergy Clin Immunol, 2008, 121: 744-749.	61	3.81	Pathogenesis、Diagnostic methods
88	Varjonen E, Petman L, Mäkinen-Kiljunen S. Immediate contact allergy from hydrolyzed wheat in a cosmetic cream [J]. Allergy, 2000, 55: 294-296.	61	2.54	Diagnostic methods 、Pathogenesis
89	Chu PT, Lin CS, Chen WJ, et al. Detection of Gliadin in Foods Using a Quartz Crystal Microbalance Biosensor That Incorporates Gold Nanoparticles [J]. J Agric Food Chem, 2012, 60: 6483-6492.	60	5	Diagnostic methods
90	Varjonen E, Vainio E, Kalimo K. Antigliadin IgE - indicator of wheat allergy in atopic dermatitis [J]. Allergy, 2000, 55: 386-391.	60	2.5	Pathogenesis
91	Houba R, VanRun P, Heederik D, et al. Wheat antigen exposure assessment for epidemiological studies in bakeries using personal dust sampling and inhibition ELISA [J]. Clin Exp Allergy, 1996, 26: 154-163.	60	2.14	Epidemiology
92	Cabanillas B. Gluten-related disorders: Celiac disease, wheat allergy, and nonceliac gluten sensitivity [J]. Crit Rev Food Sci Nutr, 2020, 60: 2606-2621.	58	11.6	Epidemiology
93	Quirce S, Diaz-Perales A. Diagnosis and Management of Grain-Induced Asthma [J]. Allergy Asthma Immunol Res, 2013, 5: 348-356.	58	5.27	Pathogenesis
94	Mamone G, Picariello G, Addeo F, et al. Proteomic analysis in allergy and intolerance to wheat products [J]. Expert Rev Proteomics, 2011, 8: 95-115.	58	4.46	Diagnostic methods
95	Yokooji T, Kurihara S, Murakami T, et al. Characterization of Causative Allergens for Wheat-Dependent Exercise-Induced Anaphylaxis Sensitized with Hydrolyzed Wheat Proteins in Facial Soap [J]. Allergol Int, 2013, 62: 435-445.	57	5.18	Pathogenesis
96	Palacin A, Varela J, Quirce S, et al. Recombinant lipid transfer protein Tri a 14: a novel heat and proteolytic resistant tool for the diagnosis of baker’s asthma [J]. Clin Exp Allergy, 2009, 39: 1267-1276.	57	3.8	Diagnostic methods
97	Franken J, Stephan U, Meyer HE, et al. IDENTIFICATION OF ALPHA-AMYLASE INHIBITOR AS A MAJOR ALLERGEN OF WHEAT-FLOUR [J]. Int Arch Allergy Immunol, 1994, 104: 171-174.	57	1.9	Pathogenesis
98	Nilsson N, Sjölander S, Baar A, et al. Wheat allergy in children evaluated with challenge and IgE antibodies to wheat components [J]. Pediatr Allergy Immunol, 2015, 26: 119-125.	55	6.11	Diagnostic methods
99	Francavilla R, Cristofori F, Castellaneta S, et al. Clinical, Serologic, and Histologic Features of Gluten Sensitivity in Children [J]. J Pediatr, 2014, 164: 463-+.	55	5.5	Clinical Manifestations
100	Ebisawa M, Shibata R, Sato S, et al. Clinical Utility of IgE Antibodies to ω-5 Gliadin in the Diagnosis of Wheat Allergy: A Pediatric Multicenter Challenge Study [J]. Int Arch Allergy Immunol, 2012, 158: 71-76.	53	4.42	Diagnostic methods

### 3.1 Year of publication

**Figure 1:**
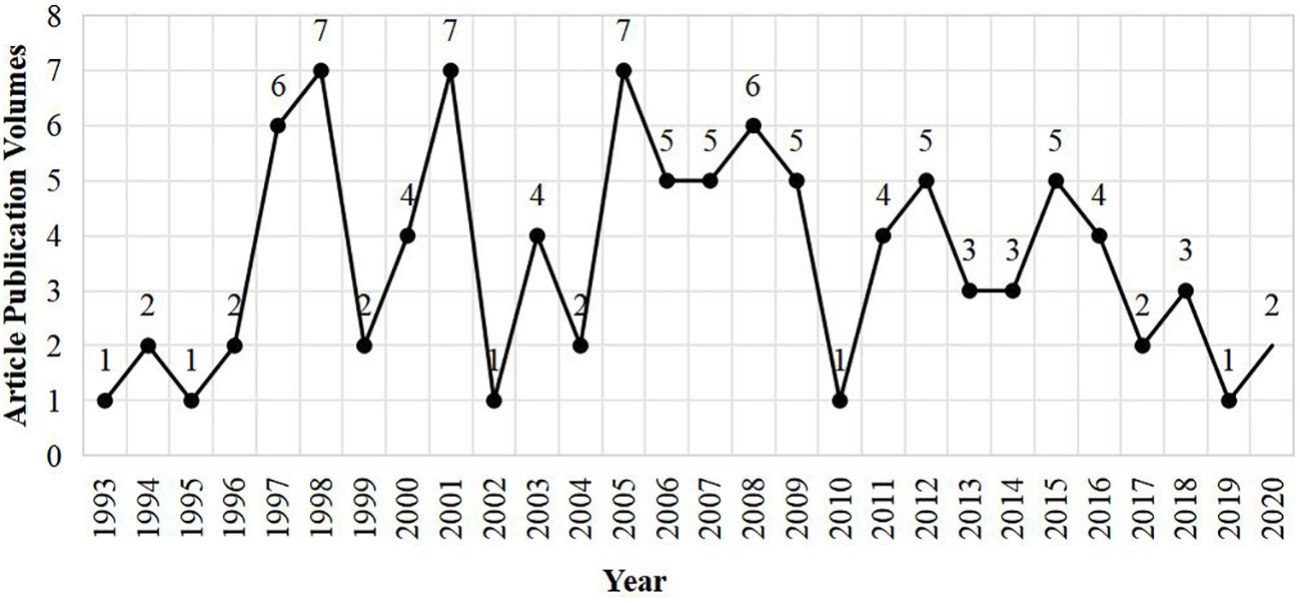
The publication volumes of the Top 100 Most Cited Allergy Research Articles each year.

The publication volumes of the top 100 most cited articles in allergy research spanning from 1993 to 2020 are illustrated in **Figure 1**. The horizontal axis indicates the years, while the vertical axis shows the number of articles published annually. This line graph allows for the observation of significant fluctuations in publication volume throughout the specified timeframe. Particularly, the years 1998, 2001, and 2005 witnessed a surge to peak publication volumes with seven articles each being recognized as the most highly cited. In contrast to these peak years, the publication volume during the intervening periods was relatively lower. Overall, the chart delineates the dynamic evolution of publication volumes over the span of nearly three decades in allergy research, serving as a visual representation of scholarly activity in this field.

### 3.2 Citations

Citation counts varied from 53 to 748. The study by Sapone et al., titled “Spectrum of gluten-related disorders: consensus on new nomenclature and classification” and published in BMC Medicine, was not only the most cited in the field of wheat allergy but also achieved the highest annual citation yield (ACY). In contrast, the least cited article, “Clinical Utility of IgE Antibodies to ω-5 Gliadin in the Diagnosis of Wheat Allergy: A Pediatric Multicenter Challenge Study” by Ebisawa M, Shibata R, Sato S, et al., appeared in the International Archives of Allergy and Immunology.

**Figure 2:**
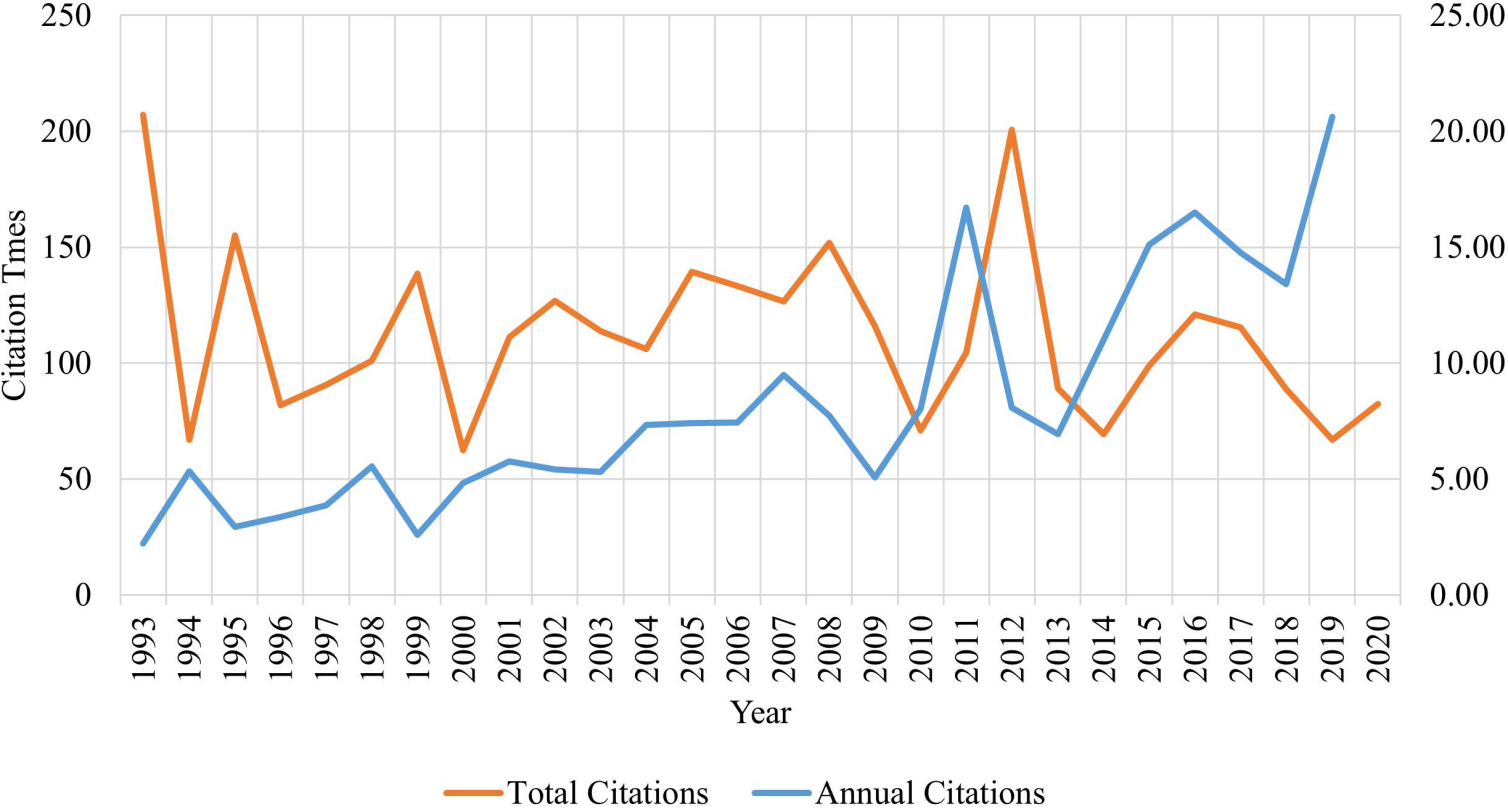
Citation times over time: total vs. annual citation times (1993-2020). The orange line represents the average total citations in different year, and the blue line represent the average annual citations of the published articles.

**Figure 2** illustrates both the annual total citations and the average citations per article. The year 1993 marked the peak for total citations, reaching 270, whereas the most cited article was published in 2020. An upward trend in the average yearly citations was consistently observed. Additionally, our analysis indicated that six articles garnered more than 200 citations each, with two in Clinical and Experimental Allergy and the others in BMC Medicine, Journal of Allergy and Clinical Immunology, Pediatrics, and Electrophoresis.

### 3.3 Journal of publication

**Figure 3:**
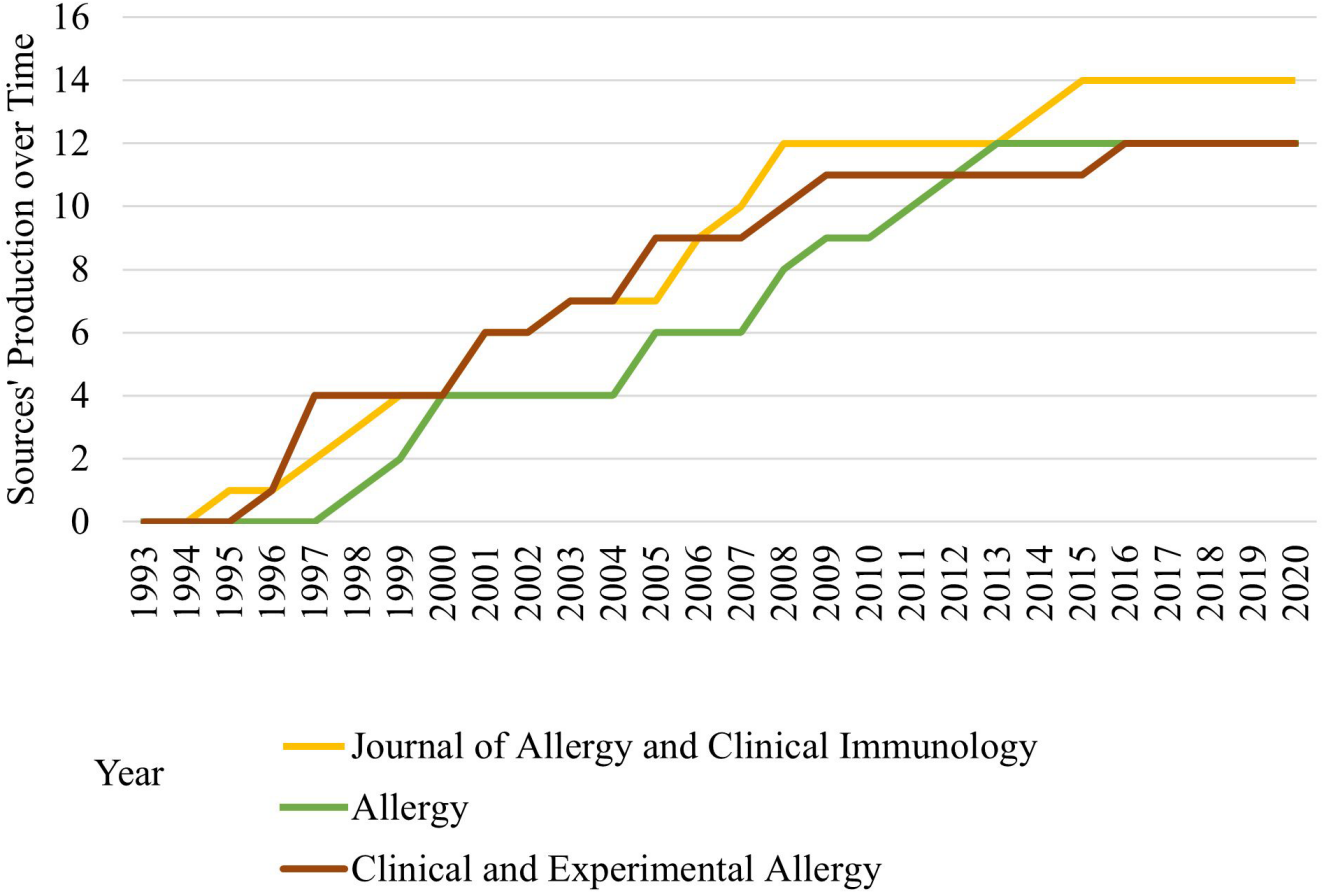
Comparative Growth in Publication Volume of Journals (1993-2020). The line graph displays the increasing publication volume for three journals over time, with “The Journal of Allergy and Clinical Immunology” showing the highest growth, followed by “Clinical and Experimental Allergy,” and “Allergy” with the smallest increase.

A total of 100 articles were disseminated across 48 distinct journals, with the Journal of Allergy and Clinical Immunology leading in publication count, contributing 14 articles. This was followed by The Journal of Allergy and The Clinical and Experimental Allergy, each with a contribution of 12 articles. **Figure 3** underscores the ascending trend in publication volumes across the three journals, notably with “The Journal of Allergy and Clinical Immunology” leading the way with the highest number of publications, indicative of its predominant role in allergy and clinical immunology research between 1993 and 2020. **Table 2** details the top 10 journals along with their impact factors and quartile scores. Out of the total articles, 65 were featured in 22 journals falling under the Q1 quartile score category. Additionally, journals with an impact factor greater than 10 published 23 of these articles.

**Table 2:** Top 10 list of journals with published articles.

Sources	Articles	Journal Impact Factor(JIF)	Quartile Score (Rank by Journal Impact Factor)	Journal Citation Indicator (JCI)	Quartile Score [Rank by Journal Citation Indicator (JCI)]
**Journal of Allergy and Clinical Immunology**	14	14.2	1	2.44	1
**Allergy**	12	12.4	1	1.91	1
**Clinical and Experimental Allergy**	12	6.1	1	0.89	1
**British Journal of Dermatology**	3	10.3	1	2.91	1
**Current Opinion in Allergy and Clinical Immunology**	3	2.8	3	0.39	3
**International Archives of Allergy and Immunology**	3	2.8	3	0.55	2
**Journal of Agricultural and Food Chemistry**	3	6.1	1	1.39	1
**Journal of Cereal Science**	3	3.8	2	0.83	2
**Allergology International**	2	6.8	1	0.88	1
**Electrophoresis**	2	2.9	3	0.62	3

### 3.4 Countries and authors

The first corresponding authors of the 100 articles were from 20 different countries. According to the list, the Japan (n=20) was the most contributing country, followed by Germany (n=17) and the USA (n=11) (**Table 3**). The highest-ranking 10 authors in the T100 cited articles were listed in **Table 4**. A noteworthy mention is Prof. Morita Eishin from Shimane University, who stands as the preeminent author in WA research, having the highest citation count for 11 of his articles. Following him is Prof. Matsuo Hiroaki from Hiroshima University, credited with 10 significant contributions.

**Table 3:** Ranking of the top 10 countries by number of articles published.

Country	Number of articles
Japan	20
Germany	17
Usa	11
Finland	9
Italy	9
Spain	8
France	4
Netherlands	4
United Kingdom	4
Australia	2

**Table 4:** Top 10 authors with the most published articles.

Corresponding author	Number of articles
Morita, Eishin	11
Matsuo, Hiroaki	10
Varjonen, Eeva	7
Diaz-Perales, Araceli	5
Kohno, Kunie	5
Palosuo, Kati	5
Quirce, Santiago	5
Alenius, Harri	4
Kalkkinen, Nisse	4
Reunala, T.	4

### 3.5 Keywords analysis, co-occurrence network, and trend topics

**Figure 4:**
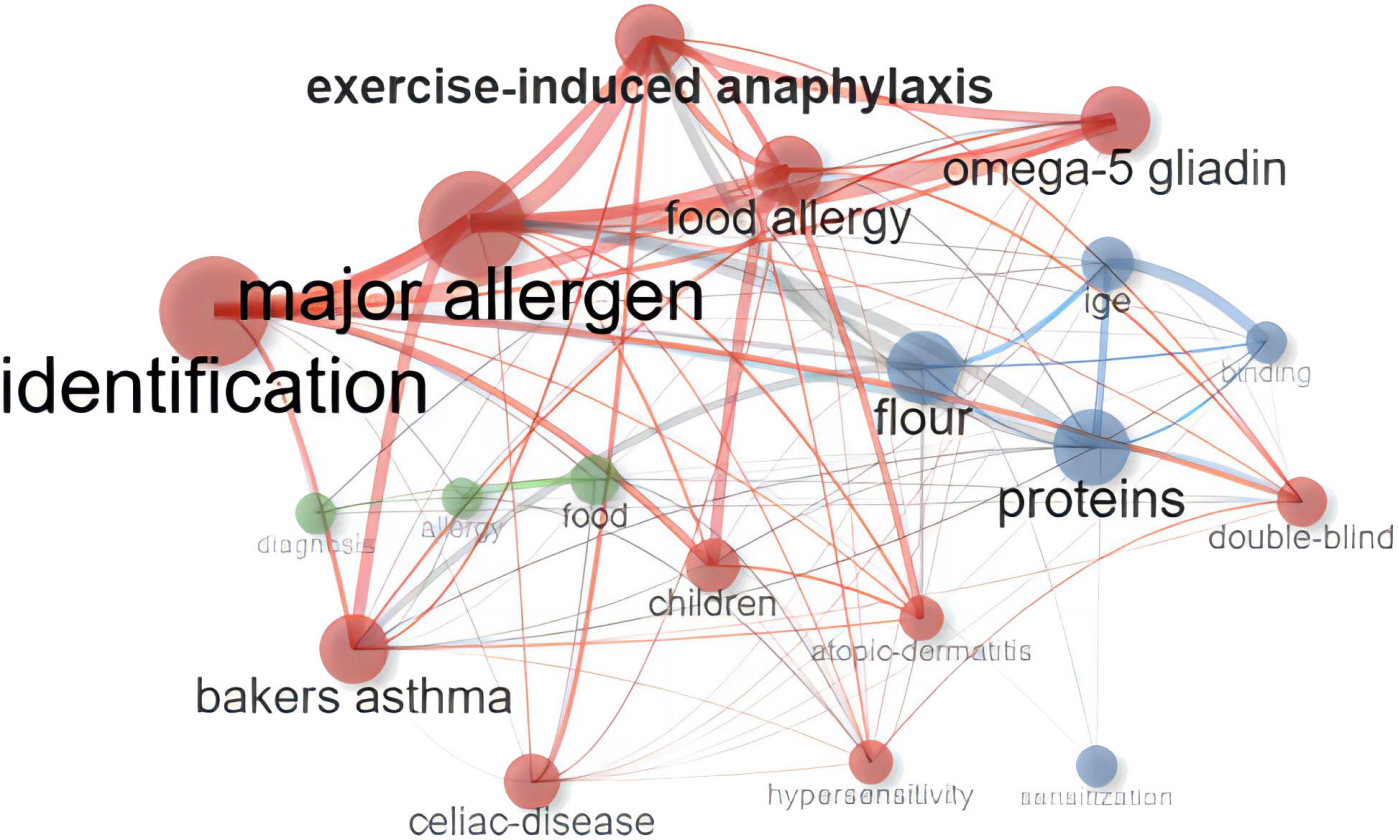
This figure presents a visualized word cloud of the most frequently used keywords within the Top 100 list. The relative font size directly corresponds to the frequency of keyword occurrences.

The analysis of authors’ most frequently used keywords is a crucial method for identifying hot topics and the focus of scholars in a particular field. The word cloud depicted in **Figure 4** illustrates that the most commonly used keywords were “identification,” “major allergen,” “celiac disease,” “food allergy,” and “omega-5 gliadin.” It is important to note that while celiac disease is not a wheat allergy (WA) disease, it often appears alongside WA in research, hence its prominent presence in the word cloud.

**Figure 5:**
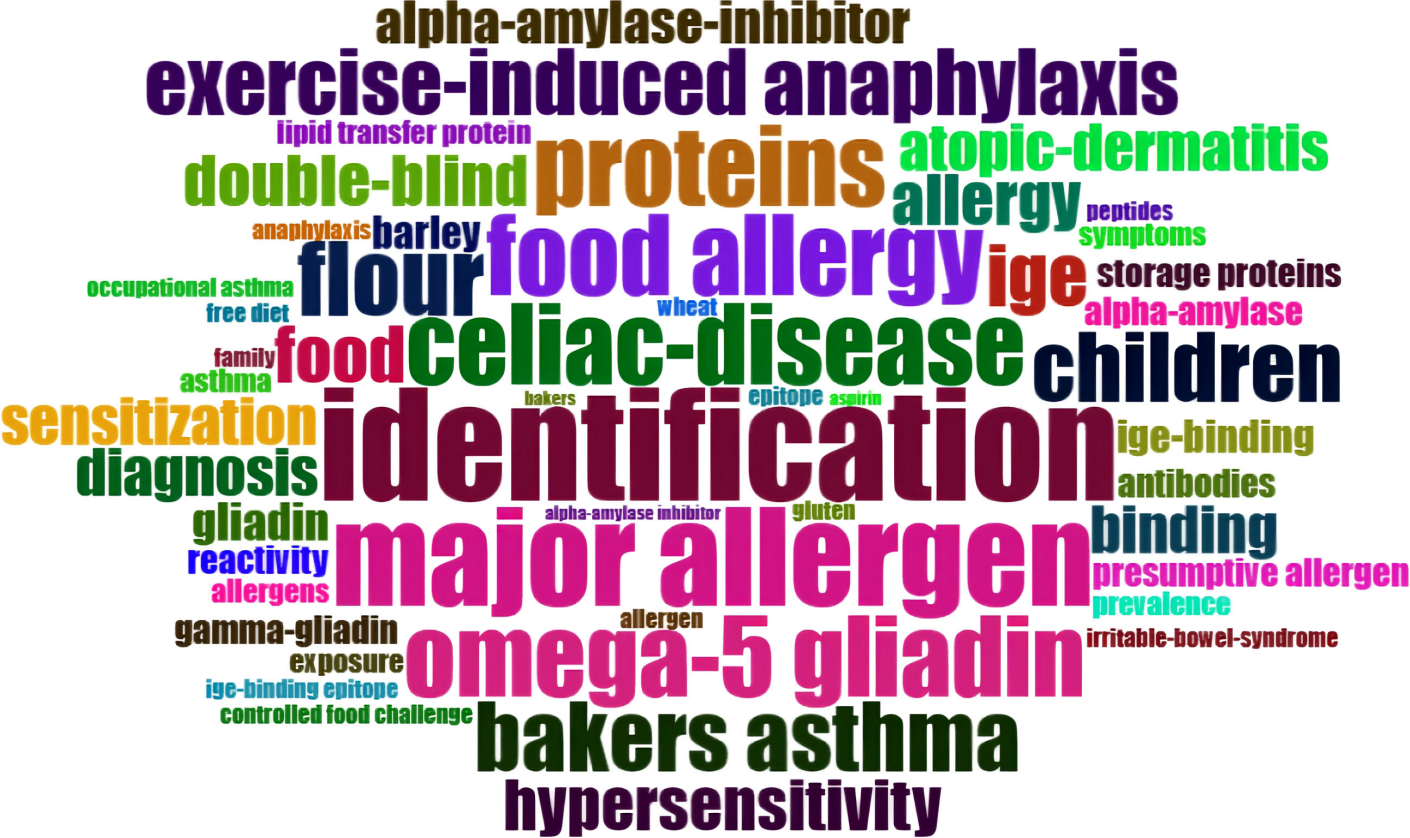
This figure depicts a co-occurrence network where the thickness of the lines signifies the strength of the relationship between keywords, with thicker lines denoting stronger associations and thinner lines indicating weaker ones. Keywords that are not interconnected by lines are indicative of an absence of established relationships.

**Figure 6:**
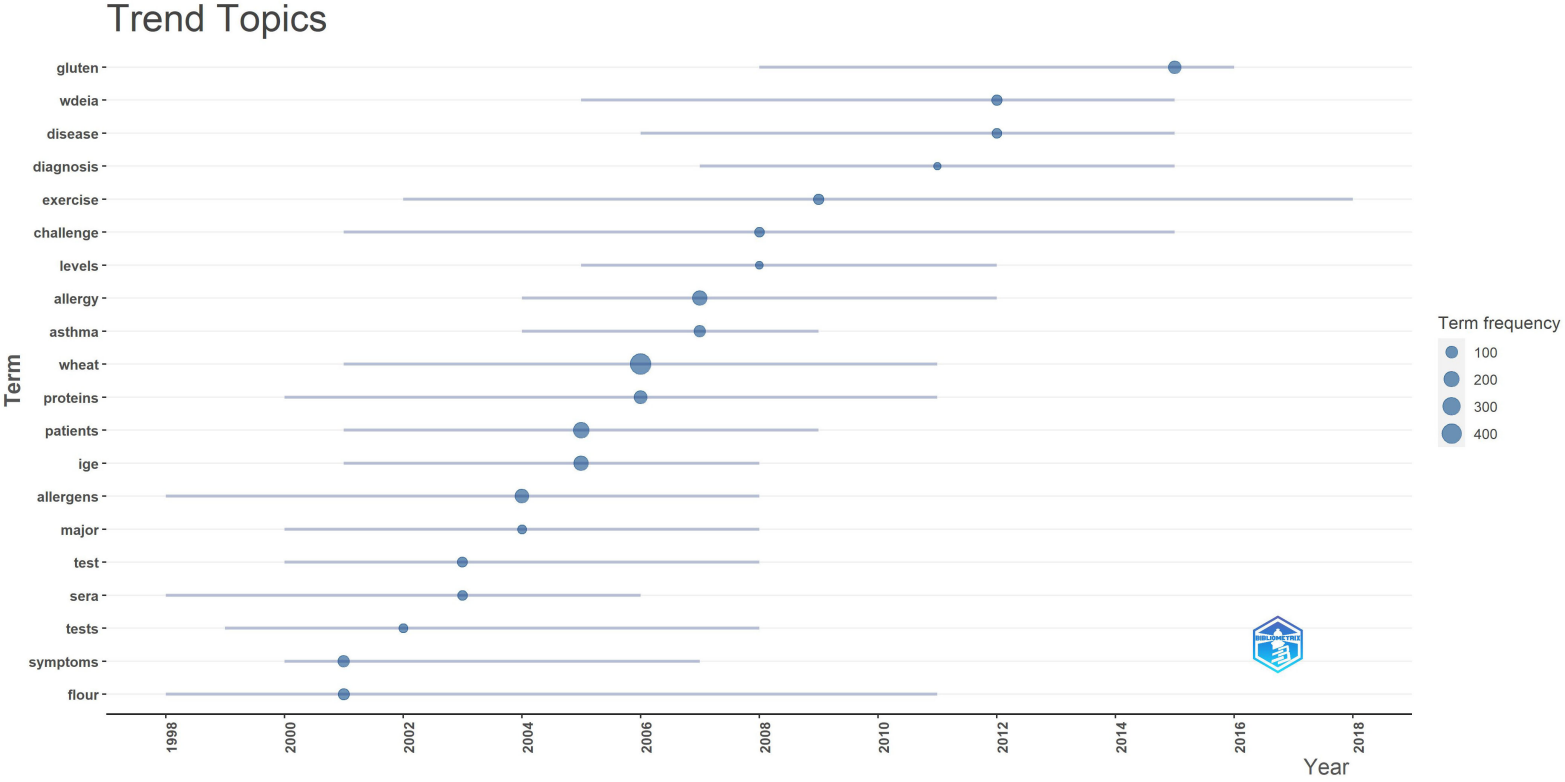
This figure illustrates the trend analysis, delineating the hierarchical organization of topics related to wheat allergy (WA) as discussed annually by scholars.

Our research within allergology entailed a comprehensive analysis of the keyword co-occurrence network, pivotal in deciphering the intricate relationships among terms commonly used in the field, as depicted in **Figure 5**. Within this network, the significance of omega-5 gliadin is particularly striking. As a critical node, omega-5 gliadin forms a nexus of connections with a multitude of other keywords, accentuating its fundamental importance in the continuum of allergy research. Trend topics analysis gave further insight into the trending topics in terms of keyword occurrences in WA literature over the years (**Figure 6**). While conducting the analysis, the following parameters were configured. The search field was set to abstract. Word minimum frequency was set to 40 and the number of words per year was set to 2. In 2006, wheat emerged as the predominant subject of discussion in allergy research. Subsequent findings revealed a marked increase in attention towards Wheat-Dependent Exercise-Induced Anaphylaxis (WDEIA), a severe, life-threatening food allergy characterized by reactions occurring post-consumption of wheat, particularly when followed by exercise within a 6-hour window, gaining prominence as a research topic from 2012 onwards.

## 4 Discussion

Wheat allergy, predominantly seen in children and potentially progressing to WDEIA in adults, is defined by an immune response to wheat proteins. The research conducted by Mari Takei et al. highlights the significance of cross-reactivity between wheat and other cereals in the effective management of wheat allergy in pediatric populations (8). Additionally, Magdalena Kraft and colleagues have identified that allergic reactions in adults often exhibit a cofactor-dependent nature and tend to be more severe than other food allergies (2). The contemporary diagnostic approaches for wheat allergy include an evaluation of clinical history, dietary assessments, and targeted allergen testing, both through skin and serological methods (9). Although there is no evidence to suggest an increasing prevalence of wheat allergy, an increase in such clinical cases has been observed. It is widely recognized that citation analysis offers an extensive overview of academic influence across journals, institutions, and authors, serving as a tool to pinpoint seminal works and journals of notable impact. While a comprehensive dissection of the top 100 most-cited articles may be beyond reach, discerning certain patterns remains feasible. Such bibliometric scrutiny not only sheds light on the prevailing themes of wheat allergy research historically but also maps the evolving trajectory of the field (10).

Bibliometric Analysis, a pivotal methodology in scientific research, specializes in quantifying the attributes of scientific literature. Its impact is multifaceted, extending beyond the mere quantification of research outcomes, such as the impact evaluation of papers or scientists via citation analysis. It encompasses the identification of emerging trends and research hotspots, exploration of academic networks, reinforcement of scientific policymaking and decision processes, optimization of research resource allocation, and the facilitation of interdisciplinary research endeavors. Consequently, Bibliometric Analysis emerges as an indispensable tool in comprehending the evolution of scientific knowledge and in shaping research policies and practices.

Particularly in niche domains like wheat allergy research, the significance of Bibliometric Analysis is magnified. It enables researchers to discern prevailing trends, pinpoint seminal studies and scholars, and uncover existing research voids and novel prospects. This dual role of deepening the comprehensive understanding of the wheat allergy research landscape and steering the trajectory of future inquiries lays a robust scientific foundation for addressing the challenges posed by wheat allergy. In essence, Bibliometric Analysis is instrumental in the realm of wheat allergy research, profoundly enhancing our comprehension of the subject and catalyzing the advancement of related research.

While the 100 top-cited articles were distributed across 48 distinct journals, a substantial concentration was observed in specific journals such as Journal of Allergy and Clinical Immunology, Allergy, Clinical and Experimental Allergy, British Journal of Dermatology, Current Opinion in Allergy and Clinical Immunology, International Archives of Allergy and Immunology, and Journal of Agricultural and Food Chemistry, which collectively accounted for half of these articles (n=50). This trend reveals that pivotal papers related to Wheat Allergy (WA) predominantly appear in a select group of leading journals, offering a comprehensive overview of the field. This pattern aligns with Bradford’s law (11), which posits the exponentially diminishing returns of broadening a search for references in scientific journals and is routinely employed to identify core journals in a specific discipline. Furthermore, our analysis indicates that the distribution of author publications adheres to Lotka’s Law (12, 13) suggesting a highly uneven distribution of scientific output among authors in the literature. This principle, established by Alfred J. Lotka (14) underscores the observation that a majority of authors typically contribute a single publication, while a minority of authors are responsible for multiple papers. Within our top 100 (T100) list, as shown in **Table 5**, merely seven authors have more than five publications, with Morita Eishin leading with 11, followed by Matsuo Hiroaki with 10, Varjonen Eeva with 7, and Diaz-Perales Araceli, Kohno Kunie, Palosuo Kati, and Quirce, Santiago each with 5. Therefore, by prioritizing a handful of premier journals and the contributions of foremost authors, allergists can efficiently stay informed about advancements in their field.

**Table 5:** Authorship distribution according to Lotka’s Law.

Documents written	Number of Authors	Proportion of Authors
1	376	0.846847
2	44	0.099099
3	12	0.027027
4	5	0.011261
5	4	0.009009
7	1	0.002252
10	1	0.002252

**Figure 7:**
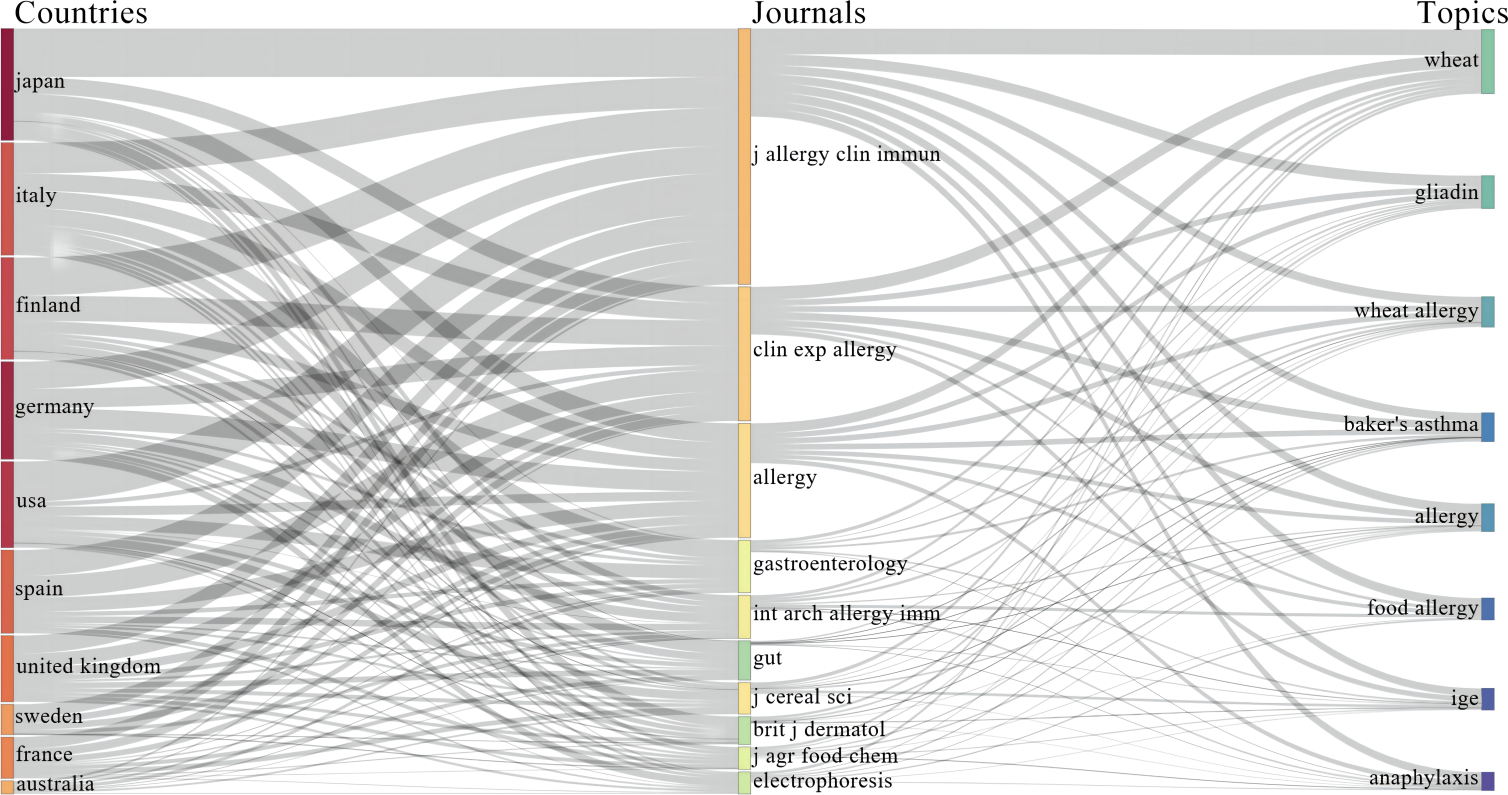
Global Distribution of Wheat Allergy Research: A Sankey Diagram Analysis. This figure is a three-column Sankey diagram that depicts the relationships between countries involved in wheat allergy research (left column), journals (middle column), and specific research topics (right column). The thickness of the lines indicates the volume of publications each country has on specific topics. For instance, Japan has a considerable number of publications in “Allergy Clinical Immunology,” while Italy has a higher publication count in “Gastroenterology.” This intuitive display assists researchers and decision-makers in rapidly identifying areas of intense research focus and potential collaborators. (The settings for the three-field Sankey diagram specify that each of the three fields—Middle, Left, and Right—displays 10 items each. The Middle Field is set to show “Cited Sources,” the Left Field displays “Countries,” and the Right Field is configured to exhibit “Keywords).

We noted that all significantly influential articles identified in our study originated from developed countries, including Japan, Italy, Finland, Germany, and the USA, as detailed in **Figure 7**. This figure presents a detailed three-field plot, effectively illustrating the interdisciplinary trends in allergy research by integrating data from various countries, relevant scientific journals, and key research terms. The plot, organized from left to right, begins with a column listing the contributing countries, thereby showcasing the geographic distribution of allergy research. The middle column connects these countries to the respective journals where the studies were published, highlighting the link between geographical and thematic elements of the research. The rightmost column focuses on specific keywords central to these publications, succinctly capturing the core themes of ongoing scholarly dialogue in this field. This visual layout not only delineates the global research landscape in allergology but also elucidates the channels of information dissemination and thematic focuses prevalent in top-tier literature. In contrast, despite a rising incidence of wheat allergy (WA) in China, as observed in our clinical practice (though not yet supported by epidemiological surveys), publications from China on this subject garner noticeably fewer citations, occasionally none (15, 16). This disparity might be attributed to China’s developing status and the consequent limited involvement of researchers from such countries, often a result of uneven global health resource allocation (17).

**Figure 8:**
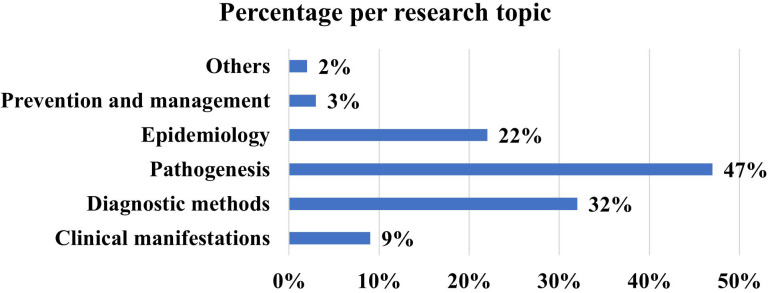
Distribution of Allergy Research Topics by Percentage in the Top-Cited List. This figure delineates the proportional distribution of scholarly focus within the field of allergy research. The horizontal bar graph illustrates that the bulk of research, nearly half at 47%, is concentrated on pathogenesis. Diagnostic methods also constitute a substantial segment, accounting for 32% of the literature. Epidemiological studies are represented as well, comprising 22%, while clinical manifestations have been the subject of 9% of the research. Management and prevention strategies are less emphasized, reflected by a mere 3%, and all other topics combined account for 2%. This visual representation underscores the predominant scholarly interest in understanding the underlying mechanisms of allergic diseases, which may facilitate the development of innovative diagnostic tools and therapeutic approaches.).

The Biblioshiny app, designed for thematic analysis in literature, utilizes keyword frequency to identify prevailing themes within selected articles. However, this approach might not always accurately capture the core content of the studies. To address this, we conducted a manual review and classification of the articles into six distinct categories: pathogenesis, diagnostic methods, management and prevention, epidemiology, and others, as illustrated in **Figure 8** and **Table 1**. Among these, the majority of the articles (n=47) focused on pathogenesis, highlighting the comprehensive research efforts to understand the complex nature of the disease. Despite the growing body of research dedicated to unraveling the sensitization mechanisms of wheat allergy, these mechanisms continue to elude clear understanding. Bibliometric analysis indicates that omega-5 gliadin is the primary wheat allergen, with medications, alcohol consumption, and exercise acting as contributing factors. This is particularly notable in Wheat-Dependent Exercise-Induced Anaphylaxis (WDEIA), where symptoms typically occur after consuming wheat products followed by activities ranging from mild, such as climbing stairs or cleaning, to intense exercises like running. The earliest article to identify gliadin as a common cause of food-dependent, exercise-induced wheat allergic reactions was “A novel wheat gliadin as a cause of exercise-induced anaphylaxis” by Palosuo K, Alenius H, Varjonen E, et al., published in the Journal of Allergy and Clinical Immunology in 1999. This study, the most cited in relation to the pathogenesis, emphasized wheat as a significant trigger for WDEIA and identified a new type of γ-like gliadin as the primary allergen. Additionally, the study highlighted the effectiveness of a gluten-free diet in managing this severe allergy, with strict avoidance remaining the primary treatment approach to date. However, it is actually omega-5 gliadin that is the major wheat allergen. In 2001, Kati Palosuo first proposed in the article “Wheat ω-5 gliadin is a major allergen in children with immediate allergy to ingested wheat” that omega-5 gliadin might be the key allergen causing wheat allergy (WA). Out of the 100 top-cited articles, 33 discuss omega-5 gliadin. These findings reflect research trends over the past 30 years and will serve as a vital foundation for future studies into wheat pathogenesis and the development of new treatment methods.

Based on extensive studies in the top 100 articles, recent advancements in the field of wheat allergy research have significantly deepened our understanding of wheat allergy. These developments include the development of low-allergenic wheat varieties (18, 19),as well as a nuanced understanding of the role of various wheat proteome components such as Tri a 14 and Tri a 19 in allergic reactions (20). Additionally, significant progress in allergen-specific IgG4 research and advancements in detection methods (21), particularly mass spectrometry (22), have also gained attention. Furthermore, global epidemiological studies have revealed various clinical characteristics of wheat allergy, including wheat-induced allergic reactions and cross-reactivity with other cereals (8). Focused studies on specific allergens such as ω-5 gliadin are crucial for understanding their impact on the severity of allergies, as evidenced by research confirming reduced IgE reactivity of WDEIA patients to wheat varieties lacking ω-5 gliadin (23). Additionally, the development of oral wheat immunotherapy (24, 25) and strategies for symptom management (26) have opened new avenues for effective treatment and management, adding new dimensions and depth to the ongoing exploration of wheat allergy mechanisms, diagnostics, and treatments.

When we examined the T100 list, another issue that merited attention came to light: daily personal care products containing wheat protein posed a potential allergenic hazard. Evidence suggested that hydrolyzed wheat protein (HWP) in these products might trigger allergic reactions. Four articles had discussed research in this area. The most cited article, published in “Allergology International,” was titled “Wheat-Dependent Exercise-Induced Anaphylaxis Sensitized with Hydrolyzed Wheat Protein in Soap.” This study investigated nine patients who developed contact urticaria after using cosmetics containing HWP. It posited that hydrolysis enhanced the allergenicity of wheat protein due to the formation of new epitopes and peptide aggregates during the hydrolysis process, which could trigger allergic reactions through both skin and digestive pathways. The second most cited article, also published in “Allergology International,” discussed wheat-dependent exercise-induced anaphylaxis (WDEIA) potentially triggered by the use of soap containing hydrolyzed wheat protein. In summary, these studies highlighted the potential risks associated with the use of hydrolyzed wheat protein in cosmetics, providing crucial insights into the understanding and management of wheat allergies, particularly the mechanisms of WDEIA.

Our research involved a meticulous bibliometric analysis of the top 100 most-referenced articles in Wheat Allergy studies, offering a crucial reference for guiding future research in this specialized area. While our study provides significant insights, it is essential to note the inherent limitations of our methodology. A notable aspect is our exclusive use of the Web of Science (WOS) database. This approach may suggest a potential bias, as employing various databases could result in different sets of top-cited articles. Recognizing this, our findings highlight the opportunity for more expansive future bibliometric analyses that incorporate multiple databases, thereby broadening the research scope and diversifying the understanding of Wheat Allergy literature.

## Data availability statement

The original contributions presented in the study are included in the article/**Supplementary Material**. Further inquiries can be directed to the corresponding authors.

## Author contributions

LW: Conceptualization, Data curation, Formal analysis, Funding acquisition, Investigation, Methodology, Software, Supervision, Visualization, Writing – review & editing. MZ: Conceptualization, Data curation, Formal analysis, Investigation, Methodology, Software, Visualization, Writing – original draft, Writing – review & editing. YH: Conceptualization, Data curation, Formal analysis, Investigation, Methodology, Software, Visualization, Writing – review & editing. TX: Conceptualization, Data curation, Formal analysis, Funding acquisition, Investigation, Methodology, Software, Supervision, Visualization, Writing – review & editing.
