# Supplementary material for: The top 100 most cited articles in the past 30 years of wheat allergy: a bibliometric analysis
Source: Front Immunol. 2024 Apr 22;15:1381130. doi: 10.3389/fimmu.2024.1381130 (PMC11070576; doi:10.3389/fimmu.2024.1381130)
Supplement: Supplementary file 1 [file DataSheet_1.docx]

Supplementary Material

1. Supplementary Figures


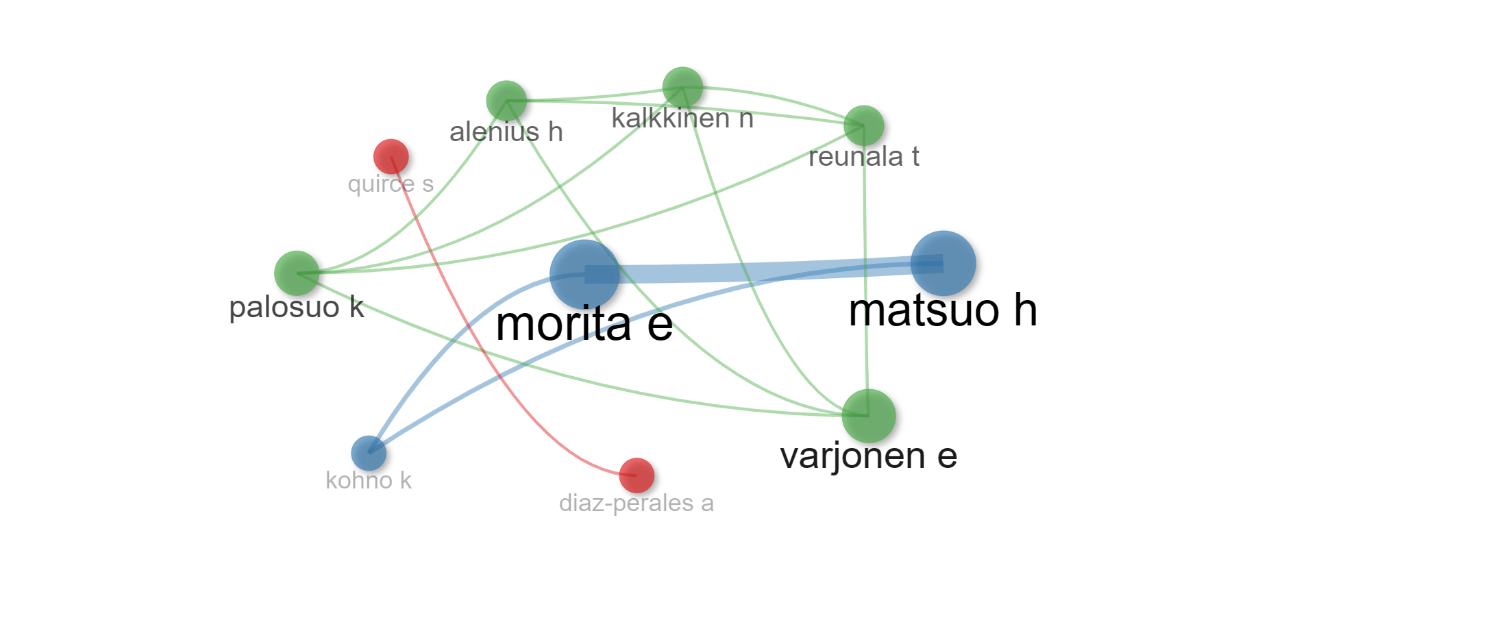


**Supplementary Figure 1.**

Collaboration Network

This figure illustrates a co-authorship network among researchers, with each node (circle) representing an author. The size of a node reflects the number of publications attributed to that author, with larger nodes signifying a more prolific publication record. Lines connecting the nodes represent co-authorship ties, with the thickness of the line indicating the volume of co-authored works.

At the center is a node labeled "Prof. Morita E," which is the largest, denoting that Prof. Eishin Morita has the highest number of publications in this network. Close in size is the node for "Prof. Matsuo H," signifying that Prof. Hiroaki Matsuo has a similarly extensive number of publications. The robust line connecting them underscores a significant collaborative history.

Other nodes, such as "Prof. Varjonen E," "Prof. Kohno K," and "Prof. Diaz-Perales A" are linked to Prof. Morita, though the connections are thinner, indicating a smaller number of collaborations.

Smaller nodes for "Prof. Alenius H," "Prof. Kalkkinen N," "Prof. Teunala T," "Prof. Quirce S," and "Prof. Palosuo K" demonstrate additional collaborative relationships and reveal a broader network of cooperation within this scientific domain, albeit with fewer publications individually than Prof. Morita or Prof. Matsuo.

This diagram effectively depicts the pattern of collaborations and individual research outputs within this scholarly community.
